# Characterization of risk factors for early ambulation in paraplegic dogs with absent pain perception undergoing decompressive surgery for thoracolumbar intervertebral disk extrusions

**DOI:** 10.3389/fvets.2024.1487105

**Published:** 2024-12-04

**Authors:** Sérgio A. Gomes, Rui Damásio Alvites, Bruna Lopes, André Coelho, Mike Targett, Jorge Ribeiro, Débora Gouveia, Ângela Martins, Artur S. P. Varejão, Ana Colette Maurício, Ana Lúcia Luís

**Affiliations:** ^1^Dovecote Veterinary Hospital, Castle Donington, United Kingdom; ^2^Departamento de Clínicas Veterinárias, Instituto de Ciências Biomédicas de Abel Salazar (ICBAS), Universidade do Porto (UP), Porto, Portugal; ^3^Centro de Estudos de Ciência Animal (CECA), Instituto de Ciências, Tecnologias e Agroambiente da Universidade do Porto (ICETA), Porto, Portugal; ^4^Associate Laboratory for Animal and Veterinary Science (AL4AnimalS), Lisbon, Portugal; ^5^Instituto Universitário de Ciências da Saúde (CESPU), Paredes, Portugal; ^6^School of Veterinary Medicine and Science, University of Nottingham, Sutton Bonington, United Kingdom; ^7^Arrábida Veterinary Hospital—Arrábida Animal Rehabilitation Center, Setúbal, Portugal; ^8^Superior School of Health, Protection and Animal Welfare, Polytechnic Institute of Lusophony, Lisbon, Portugal; ^9^Faculty of Veterinary Medicine, Lusófona University, Lisbon, Portugal; ^10^CECAV, Centre for Animal Sciences and Veterinary Studies, University of Trás-os-Montes e Alto Douro (UTAD), Vila Real, Portugal; ^11^Neurology Service, Veterinary Hospital of University of Trás-os-Montes e Alto Douro, UTAD, Vila Real, Portugal

**Keywords:** spinal surgery, nociception, spinal walker, spinal cord injury, hemilaminectomy, durotomy, French Bulldog, Dachshund

## Abstract

**Background:**

Current literature warrants surgical decompression in paraplegic dogs with absent pain perception (APP), but the rate of ambulatory dogs with APP following thoracolumbar (TL) IVDE surgery in a clinical setting remains unknown. Furthermore, the outcome of paraplegic APP French Bulldogs (FBs) is anecdotally considered poor. The aims of this study were threefold within a large population of TL-IVDE paraplegic dogs with APP undergoing decompressive surgery: (1) to characterize early spontaneous pelvic limb movement and ambulation following surgery; (2) to identify risk factors for the recovery of ambulation; and (3) to compare the outcome of FBs and Dachshunds presenting with APP.

**Methods:**

A single-center, retrospective case series of dogs with paraplegia and APP diagnosed with TL-IVDE based on CT or MRI, all undergoing surgical decompression (hemilaminectomy ± durotomy). Two main groups were defined: ambulatory and non-ambulatory. These were further characterized depending on the presence of pain perception and spontaneous movement. The outcome was obtained at 4–8 weeks postoperatively. Statistical analysis was performed comparing ambulatory and non-ambulatory dogs and comparing rates of ambulation on FBs vs. Dachshunds.

**Results:**

A total of 127 cases were included, with 77 out of 127 (60.6%) being ambulatory at recheck and 9 out of 127 (7.1%) being ambulatory despite APP. The remaining case distribution of non-ambulatory cases was: with APP (32 out of 127; 25.2%), with pain perception (5 out of 127; 3.9%), and with spontaneous movement (5 out of 127; 3.9%). Multivariate analysis revealed two negative factors for the recovery of ambulation: dogs undergoing hemilaminectomy alongside durotomy (*p* = 0.003) and dogs presenting with spinal shock (lower motor neuron signs with a lesion higher than the L3–L4 intervertebral disk) characterized by reduced/absent withdrawal reflex (*p* = 0.008). No difference was found between Dachshunds (*n* = 41, 73.2% ambulatory) and FBs (*n* = 33, 63.6% ambulatory) in terms of recovery of ambulation, with only 2 out of 33 (6.1%) FBs developing myelomalacia.

**Conclusion:**

Early recovery of ambulation alone (60.6%) and ambulation alongside pain perception (53.5%) occurred in the majority of surgically managed TL-IVDE-affected dogs with APP. Negative prognostic factors for recovery of ambulation were durotomy performed alongside hemilaminectomy, and dogs presenting with spinal shock with reduced/absent withdrawal reflexes, the latter translating to a reduced/absent withdrawal reflexes with a lesion higher than L3–L4 intervertebral disk. Finally, no indications of a worse prognosis for recovery of ambulation or a higher rate of development of myelomalacia in FBs when compared to Dachshunds were found.

## 1 Introduction

Intervertebral disk extrusions (IVDEs) are the most common cause of spinal cord injury (SCI) in dogs, causing a range of mild to severe neurological dysfunction (1–3). Selection of the most appropriate treatment modality relies on prognostic factors, the most important being the presence or absence of pain perception in the pelvic limbs. Percentages of recovery of ambulation following IVDE-induced SCI in dogs have been investigated, and current evidence suggests surgical management of paraplegic dogs with absent pain perception (APP), also termed deep pain negative or with absent nociception, with an overall recovery rate of 61% following surgery, a lower percentage than the reported 93% recovery rates when nociception is present (4). Moreover, considering the poor prognosis when pain perception is absent, euthanasia is a frequent recommendation in cases when surgery is not an option, or indeed an option when the dog does not recover following surgery (5).

Dogs without nociception following IVDE are a population of particular interest, with active research trying to identify novel therapeutic options, prognostic factors for ambulation, as well as potentiating recovery of acceptable neurological function postoperatively. Over the years, numerous studies have been conducted to investigate several factors such as the importance of offering surgical therapy to these cases in a timely manner, the outcome in terms of ambulation and urinary control, as well as factors relating to the possible development of devastating progressive myelomalacia (6). Despite a large number of studies investigating this subpopulation of dogs in the literature, most included a small population of dogs with APP, with only three reports describing populations of more than 100 dogs (7–9). Furthermore, even in larger studies, commonly found factors present in this population such as spinal shock, have not been investigated in terms of its prognostic value specifically in paraplegic APP dogs following TL-IVDE (10).

The development of paraplegia with loss of pain perception is thought to indicate a functional rather than structural SCI. Paraplegia indicates a severe, although not necessarily irreversible, loss of communication between the descending tracts arising from the central gait pattern generators in the brain and the spinal cord caudal to SCI, and APP indicates a severe, although not necessarily irreversible, loss of communication between the ascending pathways communicating the nociceptors with the somesthetic cerebral cortex (11–13). There has however been increasing recognition of the capacity of some dogs to walk or at least generate movement in the pelvic limbs, even without the return of pain perception (14–17). This has been termed spinal walking and it is thought of as reflex gait originating from local spinal cord circuits or the functioning remaining axons traversing the injury site (14). Three studies reporting on the outcome of paraplegic dogs with absent pain sensation following SCI and undergoing intensive physiotherapy revealed that 37–59% of cases developed spinal walking, in a population of dogs of TL-IVDE alone or alongside traumatic dogs with surgical or conservative management (15–17). However, it is uncertain how many dogs will become ambulatory with the return of nociception or develop ambulation with APP following thoracolumbar (TL) IVDE-induced SCI, in a clinical setting where intensive physiotherapy is not always available or indeed pursued by dog owners. In most studies with a population of >10 dogs with APP, this information was described or could only be inferred from six articles (14–19). There is a lack of a more detailed characterization of ambulation in cases following SCI due to IVDE in dogs, particularly the proportion of cases that will develop the ability to walk even without recovering pain perception.

Regarding specific breeds, the outcome of French Bulldogs without pain perception when compared to other common breeds (e.g., Dachshund) is still unknown. When looking specifically at French Bulldogs presenting without pain perception these were described as more prone to progressive myelomalacia compared to Dachshunds, with a rate of 33%, based on a small population of 15 dogs (20). Furthermore, a newer study describing 37 French Bulldogs without pain perception, has described that recovery of French Bulldogs with TL-IVDE from surgery might be poorer when compared to other breeds; however, no direct comparison was made with other breeds (21). There is limited information to suggest the assumption that French Bulldogs have a worse prognosis following a TL-IVDE with absent pain perception.

The aims of this study were 3-fold. The first one is that of characterizing early spontaneous pelvic limb movement and ambulation in a large population of paraplegic dogs with absent pain perception, undergoing surgical decompression of TL-IVDE. A second aim was to identify risk factors for recovery of ambulation for that same population, including presenting neurolocalisation and treatment options. The third was to compare the outcome of Dachshunds and French Bulldogs presenting without pain perception, following TL-IVDE.

## 2 Materials and methods

### 2.1 Animals

Ethical approval was provided by The School of Veterinary Medicine and Science at the University of Nottingham (Approval number: 4197 060824; Approval date: 08 August 2024).

Medical records of dogs, presenting to the Neurology department at Dovecote Veterinary Hospital between April 2016 and April 2024 presenting with paraplegia with absent pain perception, were retrospectively reviewed. Only dogs with complete medical records were included. Inclusion criteria included: (1) dogs presented with paraplegia with APP at presentation; (2) confirmation of a thoracolumbar IVDE (TL-IVDE) on advanced imaging (computed tomography [CT], magnetic resonance imaging [MRI]); (3) surgical decompression by means of a hemilaminectomy alone or alongside durotomy was performed; and (4) a minimum of 4 weeks and maximum of 8-weeks postoperative recheck neurological assessment. TL-IVDE encompassed the T10-L6 intervertebral disk spaces (IVDSs). Pelvic limb pain perception was tested by means of applying pressure with forceps by clamping both the medial and lateral digits of each foot as well as the base of the tail. Any repeatable behavioral response that the animal could feel the stimulus (alteration in breathing pattern, dilation of pupils or ideally turning toward the foot) was taken as pain perception being present (6). Reasons for exclusion were collected and described in detail. Surgical decompression was performed as a standard single or multiple-site hemilaminectomy alone or alongside durotomy, based on the individual choice of the clinician in charge on a case-by-case approach. All procedures were performed by ECVN board-certified veterinary neurologists or veterinary neurology residents. Following surgery, dogs were discharged with instructions to cage rest for 4–6 weeks with concurrent pain relief as required. Rehabilitation was recommended to all patients, by means of home exercises and ideally by pursuing physiotherapy in a rehabilitation center. Dogs would then be allowed to gradually resume a regular exercise routine.

Information collected from medical records included signalment, weight, duration of clinical signs (neurological dysfunction not specifically loss of pain perception or ambulatory status), functional neurolocalisation categorized as lower motor neuron (LMN, L4-S3 spinal cord segments) or upper motor neuron (UMN, T3-L3 spinal cord segments), presence of spinal shock, anatomical neurolocalisation categorized as LMN and UMN, IVDE affected disks anatomical location characterized as (thoracolumbar, midlumbar, caudal lumbar), surgical sites operated and length of hemilaminectomy, and duration of hospitalization and neurological grading at discharge. Functional neurolocalisation was based on neurolocalisation obtained at the time of presentation that may not correspond to an anatomical neurolocalisation due to the phenomenon of spinal shock (22). In functional LMN neurolocalisation, pelvic limb muscle tone was reduced and/or the withdrawal was reduced or absent in at least one of the pelvic limbs. The patellar reflex was not considered reliable on its own as defining a functional LMN neurolocalisation due to its previously reported age-related decline (23), as well as the perineal reflex. In functional UMN neurolocalisation, no abnormalities were found on segmental spinal reflexes and/or pelvic limb muscle tone. Spinal shock was defined as the reduction of the muscle tonus or the segmental reflexes caudal to the lesion identified on MRI and surgery (22). Dogs were categorized as suffering from spinal shock when presenting with LMN signs but an anatomical neurolocalisation based on MRI and surgical findings above the lumbar intumescence (presumably contained between L3-L4 and L6-L7 IVDS) (8). To further study the individual components of spinal shock, muscle tone and withdrawal reflex were classified as reduced or absent, and as normal. Anatomical UMN neurolocalisation encompassed disk material found cranial to the L3-L4 IVDS. Anatomical LMN neurolocalisation encompassed disk material found caudal to the L2-L3 IVDS. If there was an overlap between anatomical neurolocalisation (e.g., if extrusive material was found between L1 and L4), then the case would be classified as having an anatomical LMN neurolocalisation. The affected intervertebral disks identified by diagnostic imaging were recorded, covering the T10-L6 vertebral column region, and categorized as thoracolumbar (T10 through L1-2), midlumbar (L2-3 through L4-5), or caudal lumbar (L5-6 and L6-7) (24, 25). Neurological grading at discharge was obtained through a previously described 5-point scale: spinal hyperesthesia only (grade 1), ambulatory paraparesis and ataxia (grade 2), non-ambulatory paraparesis (grade 3), paraplegia with intact deep pain perception (grade 4), and paraplegia without deep pain perception (grade 5) (26).

### 2.2 Outcome: characterization of ambulation and spontaneous movement

Two main groups were defined based on the recovery of ambulation: ambulatory and non-ambulatory groups. The ambulatory group was further sub-characterized as ambulatory with pain perception and ambulatory without pain perception (termed spinal walkers). The non-ambulatory group was further sub-characterized as non-ambulatory without pain perception, non-ambulatory with pain perception, and non-ambulatory with spontaneous movement.

Recovery of ambulation was defined as a witnessed capacity of a dog to stand up and ambulate at least five independent steps at the 4–8-weeks postoperative recheck. Recovery from ambulation did not necessarily entail the return of pain sensation to the pelvic limbs. Specific information regarding the presence of nociception at the 4–8-weeks recheck was sought to distinguish ambulation with pain perception and with APP. Ambulation with APP is described as unassisted ambulation in dogs lacking pelvic limb pain perception (“deep pain negative”) (13). The non-ambulatory spontaneous movement category included dogs with the presence of spontaneous movement of the pelvic limbs without touching the animal, despite a lack of recovery of pain perception or independent ambulation. The presence of “mass reflex” or hyperreflexia, defined as alternating stepping motions of the pelvic limbs, wagging of the tail, urination, and defecation being elicited by pinching a toe or the tail was not considered as part of the latter group, as the movement was considered induced by a stimulus, and not spontaneous (27, 28).

Myelomalacia information was sought and was defined as the development of pelvic or tail lower motor neuron signs not identified on presentation, cranial migration of the caudal border of the cutaneous trunci reflex, loss of abdominal tonus eventually evolving to tetraparesis, or death by respiratory paralysis (29).

### 2.3 Statistical analysis

Exploratory statistics were performed. Analysis of data was carried out by statistical software (SPSS 29.0). Continuous variables were assessed for normality graphically and using the Shapiro–Wilk test. Median and range were reported. For the exploration of risk factors, differences in median values were compared using Mann–Whitney *U*-tests. Categorical data were reported as counts and corresponding percentages. Associations between two categorical variables were assessed using chi-squared tests. If there were fewer than five dogs in a category, Fisher’s exact test was used instead of a chi-squared test. Statistical significance was set at a *p*-value of less than 0.05.

Multivariable binary logistic regression modeling assessed the factors that are associated with recovery of ambulation. Variables at least weakly associated (*p* < 0.2) at the univariable level were considered for multivariable analysis. Multivariable model-building used a forward stepwise manual approach, focussing on presenting and treatment variables.

## 3 Results

**Figure 1:**
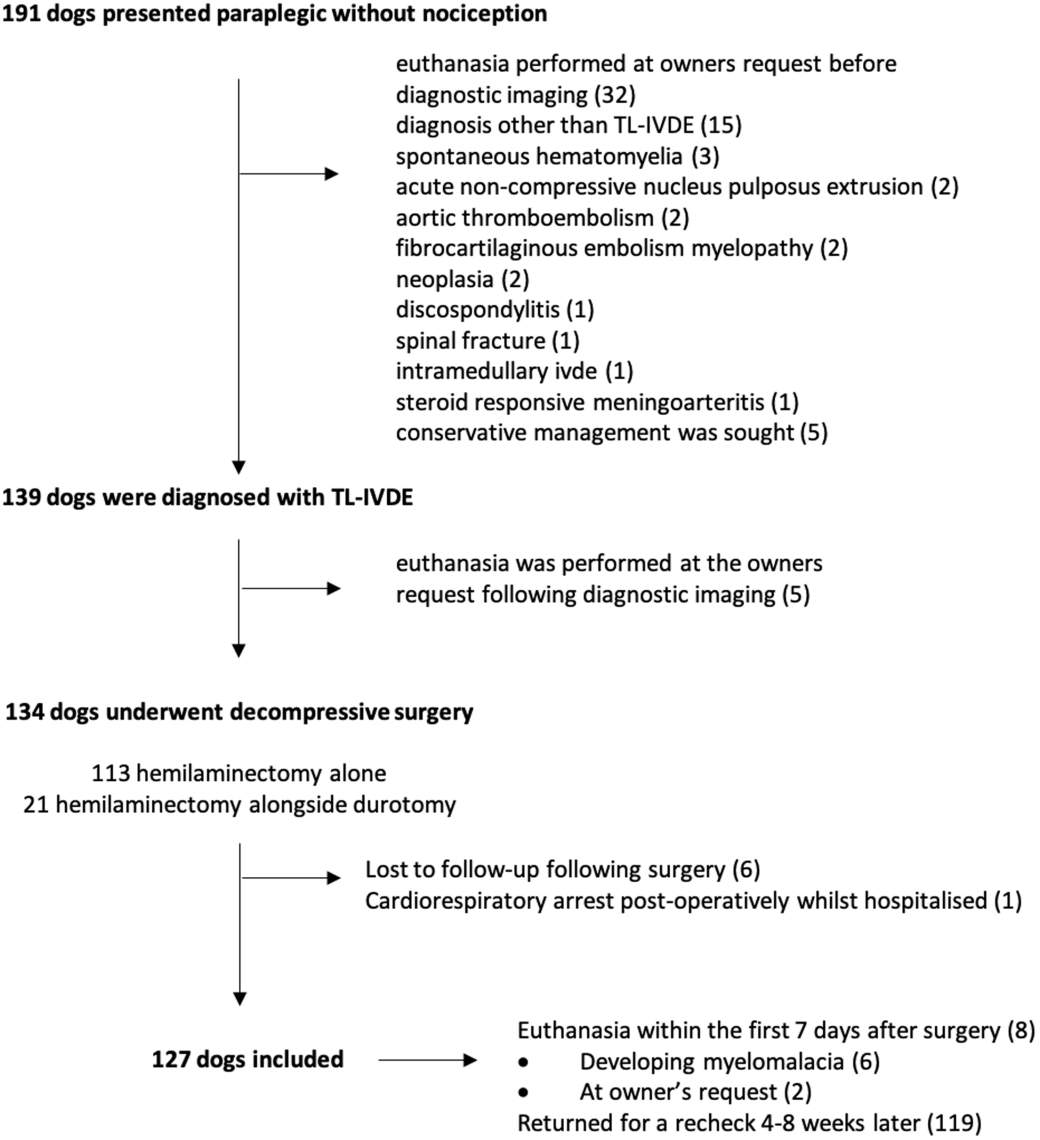
Details of the studied population and reasons for exclusion.

A total of 191 dogs presented with paraplegia without pain perception, being analyzed and categorized in terms of outcome. Details on the studied population and reasons for exclusion are shown in Figure 1. Euthanasia was performed in 16.8% of cases (32 out of 191) before diagnostic imaging was performed and in 2.6% of cases after advanced imaging but before surgery (5 out of 191). Out of the 32 cases euthanized before imaging, 4 were showing signs of progressive myelomalacia. The most common breed euthanised before diagnostic imaging was FBs (10 out of 32), none of them showing signs of progressive myelomalacia. A single case, a French Bulldog, died of cardiorespiratory arrest within the first 12-h postoperatively and was not included.

A total of 127 dogs were included in the study. The breed distribution was as follows: Dachshund (*n* = 42), French Bulldog (32), Crossbreed (16), Cockapoo (7), Border Collie (5), Cocker Spaniel (5), Shih-tzu (4), Jack Russell Terrier (3), Staffordshire Bull Terrier (3), and one instance each of Border Terrier, Cavalier King Charles Spaniel, Chihuahua, Chinese Crested Dog, Patterdale Terrier, Pekingese, Sealyham Terrier, Toy Poodle, Whippet, and Terrier. The median age at presentation was 57 months (13–156). The gender distribution was 74 males (58.3%) and 53 females (41.7%). The median weight was 9.9 kg (3.3–38). The median duration of clinical signs before presentation was 1 day (0.2–14). The median duration of hospitalization was of 4 days (range 1–15). Neurological grade at discharge revealed that 0 patients were grade 1, 4 (33.6%) grade 2, 32 (26.9%) grade 3, 17 (14.3%) grade 4, and 66 (55.5%) grade 5.

### 3.1 Outcome and characterization of early ambulation

**Figure 2:**
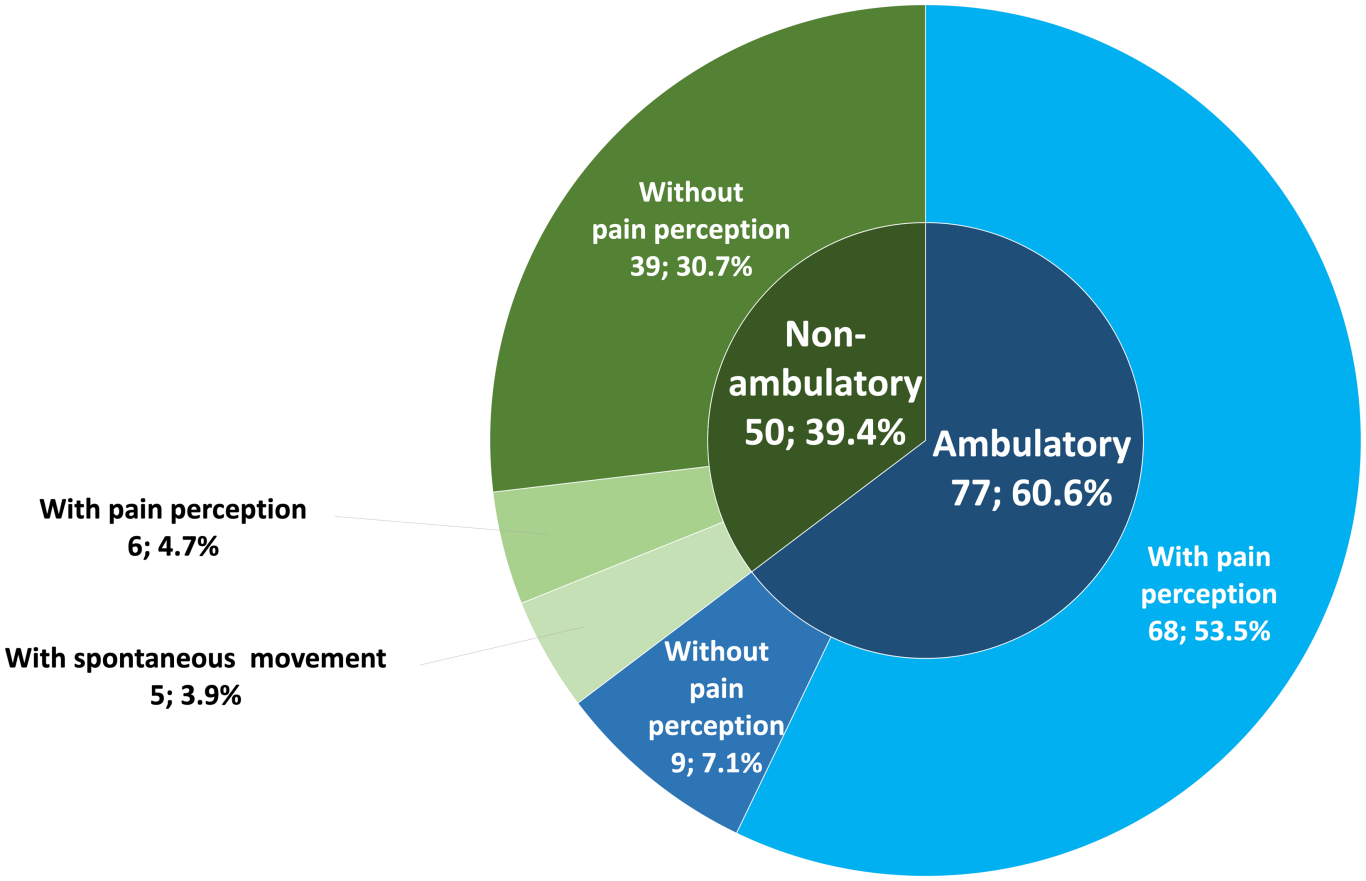
Ambulatory status and its subcategories distribution.

The outcome is detailed in Figure 2. Within the 127 included cases, a total of 8 cases were euthanized within the first 7 days postoperatively and therefore classified within the non-ambulatory group. Progressive myelomalacia developed in six cases; in one of these cases, euthanasia was intraoperative as durotomy demonstrated liquefying necrosis. Progressive myelomalacia was identified in one French Bulldog, Cockapoo, Crossbreed, Dachshund, Pekingese, and a Patterdale terrier. In two cases, that had not developed myelomalacia, euthanasia was performed at the owner’s request, and one of these cases had regained pain perception.

The majority of dogs recovered ambulation at 4–8 weeks recheck 77 out of 127 (60.6%). Within the ambulatory group, 68 out of 77 (88.3%) were ambulatory with pain perception and 9 out of 77 (11.7%) were ambulatory with APP. Within the non-ambulatory group [50 out of 127 (39.4%)], 39 out of 50 (78%) were non-ambulatory without pain perception, 6 out of 50 dogs (12%) were non-ambulatory with pain perception, and 5 out of 50 dogs (10%) were non-ambulatory with spontaneous movement. All non-ambulatory cases with spontaneous movement also had APP. All of the ambulatory dogs with APP or non-ambulatory spontaneous movement dogs were less than 15 kgs. Dogs ambulatory with APP were Dachshund (*n* = 3), French Bulldog (2), Cocker Spaniel, Crossbreed, Jack Russell Terrier, and Sealyham Terrier (1 of each).

### 3.2 Statistical analysis of risk factors of ambulation and FBs and dachshunds comparison

Detailed signalment and features of the ambulatory and non-ambulatory groups and their comparison are described in Table 1. No risk factors regarding signalment were found to be related to outcomes.

**Table 1:** Univariate statistical comparison between ambulatory and non-ambulatory dogs at 4–8 weeks following a thoracolumbar intervertebral disk extrusion (TL-IVDE) and absent pain perception (APP).

Variable	Category	Non-ambulatory	Ambulatory	*p*-value
Total number of cases (*n*, %)	–	50 (39.4%)	77 (60.6%)	
Age at onset (months)	–	Median 57.5 (range 16–99)	Median 57 (range 13–156)	0.78
Gender (*n*)	Male	29	45	0.961
	Female	21	32	
Neutered status (*n*)	Yes	35	43	0.109
	No	15	34	
Weight (kg) (*n*)		Median 9.95 (range 4–19.1)	Median 9.9 (3.3–38)	0.431
Weight categorized (*n*)	Dogs <15 kg	43	62	0.425
	Dogs ≥15 kg	7	15	
Breed (*n*)	Border Terrier	1	0	0.227
	Border Collie	0	5	
	Cavalier King Charles Terrier	1	0	
	Chihuahua	0	1	
	Chinese Crested Dog	0	1	
	Cockapoo	3	4	
	Cocker Spaniel	3	2	
	Crossbreed	10	6	
	Dachshund	12	30	
	French Bulldog	11	21	
	Jack Russell Terrier	2	1	
	Patterdale Terrier	1	0	
	Pekingese	1	0	
	Sealyham Terrier	0	1	
	Shih Tzu	2	2	
	Staffordshire Bull Terrier	1	2	
	Toy Poodle	1	0	
	Whippet	1	0	
	Yorkshire Terrier	0	1	
Duration of clinical signs (days)	–	Median 1.5 (range 0.25–14)	Median 1 (range 0.2–14)	0.702
Functional neurolocalisation (at presentation) (*n*)	T3–L3 (UMN)	12	32	**0.042**
	L4–S3 (LMN)	38	45	
Anatomical neurolocalisation (based on imaging and surgical findings) (*n*)	T3–L3 (UMN)	36	56	0.929
	L4–S3 (LMN)	14	21	
Muscle tonus at presentation	Normal	20	27	0.574
	Reduced	30	50	
Withdrawal reflex at presentation	Normal	13	40	**0.004**
	Reduced/absent	37	37	
Spinal shock (lower motor neuron signs with lesion higher than L3–L4) (*n*)	Present	24	24	0.056
	Absent	26	53	
Spinal shock and muscle tonus	Normal	38	63	0.427
	Reduced	12	14	
Spinal shock and withdrawal reflexes	Normal	26	59	**0.004**
	Reduced/absent	24	18	
Duration of hospitalization (days)	–	Median 4 (range 1–15)	Median 4 (range 1–15)	0.405
Grading at discharge (*n*)	1	0	0	**<0.001**
	2	0	4	
	3	1	31	
	4	1	16	
	5	40	26	
Euthanasia before discharge		8	0	
Region IVDE (*n*)	Thoracolumbar (T9–L2)	32	45	0.44
	Midlumbar (L2–L5)	9	23	
	Lumbosacral (L5–S1)	1	1	
	Multifocal (across multiple regions)	8	8	
Surgery: length of intervertebral disk spaces open during hemilaminectomy (*n*)	1	18	45	0.063
	2	15	17	
	3	11	7	
	4	5	8	
	5	1	0	
Surgery: single space hemilaminectomy vs. multiple space hemilaminectomy (*n*)	Single space	18	45	**0.013**
	Multiple spaces	32	32	
Surgery: hemilaminectomy alone vs. hemilaminectomy alongside durotomy (*n*)	Hemilaminectomy	35	71	**0.001**
	Hemilaminectomy alongside durotomy	16	6	
Surgery: side of surgery (*n*)	Left	24	44	0.313
	Right	26	33	

Bold in the *p*-value column indicates statistical significance (*p* < 0.05).

Univariate statistical analysis significance analysis (*p* < 0.05) indicated three positive and three negative factors for recovery of ambulation (Table 1). Positive factors included the presence of a functional UMN neurolocalisation at presentation (*p* = 0.042), undergoing a single-site hemilaminectomy instead of a multisite hemilaminectomy (*p* = 0.013), and the presence of pain perception or spontaneous movement at the time of discharge (*p* = 0.001) [neurological grade < 5 at discharge, discharge occurred at a median of 4 days, range 1–15]. Negative factors included the presence of withdrawal reflex reduced/absent at presentation (*p* = 0.004), spinal shock and withdrawal reflex reduced/absent at presentation dogs (*p* = 0.004), and dogs undergoing hemilaminectomy alongside durotomy instead of hemilaminectomy alone (*p* = 0.001).

Multivariate statistical analysis indicated two statistically significant negative factors for recovery of ambulation (Table 2): (1) dogs undergoing hemilaminectomy alongside durotomy were approximately five times less likely to recover ambulation compared to those undergoing hemilaminectomy alone [odds ratio (OR) 0.202 (95% CI: 0.070, 0.584); *p* = 0.003]; and (2) dogs demonstrating spinal shock at presentation and absent withdrawal reflex were approximately three times less likely to recover ambulation compared to those who experience spinal shock with a normal withdrawal reflex [OR 0.339 (95% CI: 0.152, 0.752); *p* = 0.008]. This multivariate model was performed with the removal of the overwhelming effect of the variable return of pain perception or movement at the time of discharge, to better assess neurological features at presentation and treatment options.

**Table 2:** Multivariate statistical comparison between ambulatory and non-ambulatory dogs at 4–8 weeks following a thoracolumbar intervertebral disk extrusion and absent pain perception.

Variable	OR (95% CI)	*p*-value
Surgery: hemilaminectomy alongside durotomy instead of hemilaminectomy alone	0.202 (0.070, 0.584)	0.003
Spinal shock at presentation with reduced/absent withdrawal reflexes	0.339 (0.152, 0.913)	0.008

The *p*-value column indicates statistical significance (*p* < 0.05).

Univariate comparison between Dachshunds and French bulldogs revealed no statistical difference in terms of recovery of ambulation (*p* = 0.378) (Table 3). A total of 73.2% (30 out of 41) of Dachshunds and a total of 63.6% (21 out of 33) of French Bulldogs recovered from ambulation. A total of 10% (3 out of 30) of ambulatory Dachshunds and 9.5% (2 out of 21) of ambulatory French Bulldogs were ambulatory with APP.

**Table 3:** Comparison between Dachshunds and French Bulldogs non-recovering or recovering ambulation following a thoracolumbar intervertebral disk extrusion and loss of pain perception.

Variable	Total	Non-ambulatory	Ambulatory	*p*-value
Dachshunds (*n*, %)	41	11, 26.8%	30, 73.2%	0.378
French Bulldogs (*n*, %)	33	12, 36.4%	21, 63.6%	

The *p*-value column indicates statistical significance (*p* < 0.05).

## 4 Discussion

In a large population of paraplegic dogs with APP following TL-IVDE, we aimed to characterize early ambulation, risk factors for return of ambulation, and the outcome of French bulldogs compared to Dachshunds.

Previous literature describing dogs with APP has used several distinct definitions for the return of ambulation, with the majority not explicitly distinguishing between the return of ambulation with the return of pain sensation and the development of spinal walking. Definitions included different combinations of recovery of pain perception, voluntary urinary function, and the ability to walk was sometimes defined as a dog able to give at least 10 steps without falling or not defined in any particular way (5, 13).

Our overall ambulation rate of 60.6% was generally compatible with previous reports (4). When looking specifically at cases recovering both ambulation and pain perception within 8 weeks from surgery, we found this to represent 53.5% of cases. This percentage is slightly higher than previous reports describing over 100 TL-IVDE-affected dogs, where recovery was reported as 46.6% (116 dogs, recovering ambulation and deep pain sensation) (9), 52% (211 dogs, recovering ambulation and deep pain sensation) (7), and 48.3% (178 dogs, recovering ambulation with or without deep pain sensation) (8).

Spinal walking is described as a reflex gait originating from local spinal circuitry, in combination with residual or recovered supraspinal connections traversing the injury site (13, 14). We opted not to use the term spinal walking to define our population that recovered ambulation, as studying a short follow-up time (4–8 weeks) underscores the fact that some of these dogs might not be true spinal walkers, having preserved subpial descending motor tracks. Ambulatory dogs with APP represented 7.1% (9 out of 127) of our total population and 11.7% of dogs recovering from ambulation (9 out of 77). In previous studies describing paraplegic dogs undergoing hemilaminectomy in a clinical setting, the proportion of ambulatory dogs with APP could be described or inferred in two studies describing a total of 32 and 64 dogs specifically with TL-IVDE undergoing decompressive surgery and represented 14 and 11% of dogs (both with a minimum of 6 months follow-up), respectively (14, 19). This is marginally superior to the percentage of ambulatory dogs with APP in our study, although both studies reported a smaller number of cases, with longer follow-up time after surgery. Nonetheless, if in addition to the ambulatory dogs with APP, we consider the dogs with spontaneous movement, then we would be more approximate to the percentages found by those previous studies. Dogs with spontaneous movement but no ambulation have also been identified as 3 out of 211 (1.4%) in one previous study (7). A larger number of dogs without pain perception developing spinal walking has been described, when dogs underwent intensive neurorehabilitation. When spinal walking was studied in long-term paraplegic dogs with absent pain sensation following SCI as a whole, 58–59% of cases developed spinal walking (15, 16). In another study describing specifically dogs with loss of pain perception following TL-IVDE-induced SCI undergoing intensive neurorehabilitation, 37.2% of persistently deep pain-negative dogs developed spinal walking (17). Literature seems to indicate that a smaller percentage of heavier dogs will be able to develop spinal walking (15, 16), a finding supported by our findings, where none of the dogs >15 kg developed ambulation with APP or spontaneous movement within the initial 8 weeks post-surgery, despite many nonetheless recovering ambulation with pain perception.

No factors regarding signalment were found to be related to outcome. The need for a more extensive hemilaminectomy in more than one intervertebral disk space was initially significantly correlated with a poorer prognosis in univariate but not in multivariate analysis, with 71.8% (45 out of 63) of cases with a single-site hemilaminectomy recovering ambulation vs. 40.6% (26 out of 64) when hemilaminectomy had to be performed over multiple spaces. The longer extent of intramedullary hyperintensities has been reported in the literature to be related to the development of progressive myelomalacia (6), but the extent of the compressive disk material has not been identified as a predictor of outcome in dogs with APP. TL-IVDE associated with extensive epidural hemorrhage has also not been reported to lead to a worse prognosis (30). Approximately 40% of cases undergoing a more extensive hemilaminectomy recovered ambulation, emphasizing that the need for extensive hemilaminectomy should not be a further reason to euthanize these patients following advanced imaging. The functional but not the anatomical T3–L3 neurolocalisation was also found to be positively related to ambulation in univariate analysis, but not found significant in multivariate analysis. This is in agreement with previous literature, where no difference in ambulation status of dogs with IVDE was found, regardless of a T3-L3 vs. L4-S3 neurolocalisation (6, 31).

Spinal shock in dogs describes the occurrence of lower motor neuron-like signs (hyporeflexia, areflexia, or loss of muscle tone) caudal to severe spinal cord injury thought to be secondary to a sudden interruption of descending supraspinal input (22, 27). This phenomenon effectively corresponds to a mismatch in the presumed neurolocalisation at the time of presentation (L4-S3 spinal cord segments) and the effective localisation of a lesion (T3-L3 spinal cord segments), as confirmed by diagnostic imaging and surgery. In people, spinal shock typically occurs in acute transverse SCI, being rare in slowly progressing lesions (32). The same seems to occur in dogs, with spinal shock having been identified frequently in dogs with fibrocartilaginous embolism myelopathy (FCEM) or acute non-compressive nucleus pulposus extrusion (ANNPE), likely due to their typically peracute onset of clinical signs (33). Spinal shock is correlated with prognosis in human medicine, its presence indicates a poorer prognosis than equivalent spinal cord injuries without spinal shock, due to the rapid onset of injury. Furthermore, earlier return of reflexes suggests a better prognosis for patients with comparable injuries and spinal shock (32). In dogs, spinal shock *per se* has not been found to have a prognostic value for the recovery of ambulation, having been associated with the development of fecal incontinence and with a slower time to ambulation in ANNPE (27, 33–36). Nonetheless, the specific prognostic value of spinal shock in paraplegic APP dogs following TL-IVDE had not been explored previously.

Interestingly, the presence of spinal shock at presentation was not found to be a negative factor as per previous studies; however, spinal shock alongside reduced or absent withdrawal reflexes was identified as a negative predictive factor for the recovery of ambulation in our population. Following our methods, this means that dogs presenting with reduced/absent withdrawal reflexes with a lesion higher than the L3-L4 IVDS had a worse prognosis. This could be an indication that the mechanisms underlying this syndrome, might have a more lasting effect on the spinal cord, and indeed the future ambulatory capacity of these cases. A similar finding was described in a previous study studying the effect of durotomy following severe SCI in dogs: when a mismatch between a T3-L3 neurolocalisation and reduced or absent pelvic limb reflexes (patellar, flexor, or both) was found, a smaller percentage of dogs recovered ambulation than when the mismatch was not present (37). Despite this novel negative association with ambulation, it is important to interpret the results with caution, as a spinal shock as a clinical syndrome could be an indication of the sudden reversible interruption of descending supraspinal input, but could also be indicative of inflammation/oedema extending from the injury site into lumbosacral intumescence or signs consistent with (descending) myelomalacia (37). The different processes masked underneath the term spinal shock could have different consequences in ambulation. The findings in our study indicate a possible prognostic role of reduced/absent withdrawal reflexes in APP dogs with a lesion higher than the L3-L4 IVDS, a role that should be investigated further in future prospective studies, in dogs sustaining severe SCI leading to APP. The individual components of spinal shock, such as the reflexes or muscle tone, could be investigated prospectively in future studies, to determine if these results would be repeatable and have a role in the prediction of outcomes in cases with severe SCI.

In our population, when durotomy was performed, dogs were significantly less likely to recover ambulation in both univariate and multivariate statistical analyses. Recent literature has explored the usefulness of durotomy, as possibly preventing the development of myelomalacia or potentially leading to a higher number of dogs recovering ambulation (9, 37), with an even more recent study concluding that durotomy is ineffective in improving functional outcomes for severe acute thoracolumbar SCI in dogs (38). As performing durotomy was based on a case-based individual clinical decision, it would in principle be difficult to infer if the procedure was causative of a worse prognosis. However, taking into consideration that durotomy has been previously shown to be a safe procedure (9, 37), it is likely that cases that underwent durotomy were those perceived to be the worst affected cases by the clinician. This subjective individual clinical assessment would need to be investigated further in future studies on the decision-making process that underlies the use of durotomy in specific cases.

Looking specifically at the population of French Bulldogs, the recovery rate of ambulation (63.6%.) was statistically not different from Dachshunds (73.2%). This contradicts the previous literature suggesting that the recovery of French Bulldogs with TL-IVDE from surgery is lower than that of other breeds, with reported rates of 33% (within 15 French Bulldogs) and 38% (within 37 French Bulldogs) (20, 21). Previous studies have demonstrated that TL-IVDE occurs at a younger age, more caudally in the vertebral column, and with greater neurological severity in French Bulldogs when compared to Dachshunds (20). Furthermore, evidence exists that the recurrence of IVDE can be expected in more than half of French Bulldogs (39). Considering these identifiable differences, the aforementioned features may have contributed to the general expectation that French Bulldogs might have a worse prognosis than other breeds. Even within our population, the most common breed euthanized even before advanced imaging was French Bulldogs. Other possible causes for this include the higher incidence of caudal IVDE in this breed which can carry a worse prognosis (20, 31), the high rate of respiratory compromise in hospitalized patients (40), the high prevalence of neurological disease in general (41), and high rate of recurrence of IVDE (39). Furthermore, only 2 out of 33 (6.1%) French Bulldogs developed myelomalacia in our study population, a considerably lower figure than previously reported (20). Our findings lend support to the notion that paraplegic with APP French Bulldogs undergoing surgery for TL-IVDE have no worse prognosis for ambulation than other breeds such as the Dachshund, and that surgery could be offered to owners and attempted in French Bulldogs, as much as in any other breed.

In our population, euthanasia was performed in 19.4% of cases, before or just after diagnostic imaging, and was performed based on poor prognosis, on a large proportion of dogs. The absence of pain perception has been related to a poor prognosis in other myelopathies besides TL-IVDE, with an even worse prognosis in cases where surgery is not an option such as in acute non-compressive nucleus pulposus extrusion or fibrocartilaginous embolism (13, 14, 36). When looking specifically at the number of cases recovering ambulation in TL-IVDE, regaining pain perception, or becoming ambulatory with APP, it seems consistent in both our population and the literature that more than 60% of cases can recover. This is an important discussion to have with owners, particularly when prepared and willing to offer the nursing care and early rehabilitation follow-up needed in these cases. Nonetheless, the quality of life of both owner and dog relies on several other factors, such as neurogenic bladder, and urinary or fecal incontinence, that has not been investigated in this study (23, 31, 42).

The limitations of this study include its retrospective nature. Both ambulation with the return of pain sensation and the development of spinal walking are time-dependent processes (13), so the short follow-up in this study might mean that a higher proportion of dogs could recover ambulation months or even years after initial SCI. The retrospective nature of this study precluded the clear definition of ambulation, which in a prospective scenario can be better defined, for instance as the ability to walk 10 steps without assistance (37). Spinal shock as a syndrome and umbrella term might include cases with a transient interruption of descending supraspinal input, but also cases with an inflammation and/or oedema that extended from the injury site into the lumbosacral intumescence (37). Other factors important for this subset of patients’ quality of life, such as urinary and fecal incontinence have also not been investigated. A single-institution study meant that the number of cases was naturally limited, but it also meant that the procedures and clinical recommendations were likely more standardized than in a multicentre study. The statistics analysis in this study was considered exploratory, as our data are retrospective.

In conclusion, early recovery of ambulation alone (60.6%, 77 out of 127) and ambulation alongside pain perception (53.5%, 68 out of 127) occurred in more than half of surgically managed TL-IVDE-affected dogs without pain perception at presentation. Early ambulation despite APP developed in 7.1% of cases, and there is the potential that in the long term the percentage of dogs recovering ambulation after TL-IVDE with loss of pain perception could be even higher than the currently reported ~60% of cases, particularly with intensive rehabilitation. Negative prognostic factors for the recovery of ambulation were found when durotomy was performed alongside hemilaminectomy and in dogs demonstrating spinal shock alongside reduced/absent withdrawal reflexes at presentation translated to reduced/absent withdrawal reflexes in APP dogs with a lesion higher than the L3-L4 IVDS. Finally, and contrary to previous literature, we have not found indications of a worse prognosis for the recovery of ambulation or even a higher rate of the development of myelomalacia in French Bulldogs when compared to Dachshunds. Considering this study was based on retrospective data, future prospective studies would be necessary to confirm our findings, which should be taken with caution.

## Data Availability

The raw data supporting the conclusions of this article will be made available by the authors, without undue reservation.
